# Rescue procedure for isolated dystonia after the secondary failure of globus pallidus internus deep brain stimulation

**DOI:** 10.3389/fnins.2022.924617

**Published:** 2022-08-18

**Authors:** Suzhen Lin, Lingbing Wang, Yimei Shu, Shunyu Guo, Tao Wang, Hongxia Li, Chencheng Zhang, Bomin Sun, Dianyou Li, Yiwen Wu

**Affiliations:** ^1^Department of Neurology & Institute of Neurology, Ruijin Hospital, Affiliated With Shanghai Jiao Tong University School of Medicine, Shanghai, China; ^2^Department of Neuro-Oncology, Beijing Tiantan Hospital, Capital Medical University, Beijing, China; ^3^Department of Neurosurgery, Center for Functional Neurosurgery, Ruijin Hospital, Affiliated With Shanghai Jiao Tong University School of Medicine, Shanghai, China

**Keywords:** rescue procedures, deep brain stimulation, dystonia, globus pallidus internus, subthalamic nucleus, pallidotomy

## Abstract

**Introduction:**

Globus pallidus internus (GPi) deep brain stimulation (DBS) is widely used in patients with dystonia. However, 10–20% of patients receive insufficient benefits. The objectives of this study are to evaluate the effectiveness of bilateral subthalamic nucleus (STN) DBS along with unilateral posteroventral pallidotomy (PVP) in patients with dystonia who experienced unsatisfactory GPi-DBS and to address the reported rescue procedures after suboptimal DBS or lesion surgery in dystonia patients.

**Methods:**

Six patients with isolated dystonia who had previously undergone bilateral GPi-DBS with suboptimal improvement were included. Standardized assessments of dystonia using the Burke-Fahn-Marsden Dystonia Rating Scale (BFMDRS) and quality of life using SF-36 were evaluated before surgery and 1, 6 months, and last follow-up (LFU) after surgery. STN bilateral OFF (bi-OFF), unilateral ON (uni-ON), and bilateral ON (bi-ON) states were recorded at LFU. Specific items were used to find publications published before 10 April 2022 regarding rescue procedures after suboptimal DBS or lesion surgery in patients with dystonia for reference. Eleven original studies including case reports/series were identified for discussion.

**Results:**

Substantial clinical benefits were achieved in all six patients. Significant amelioration was achieved during the 1-month (6.5 ± 7.45; *p* = 0.0049), 6-month (5.67 ± 6.3; *p* = 0.0056) follow-ups, and at LFU (4.67 ± 4.72; *p* = 0.0094) when compared with the baseline (LFU of GPi DBS with on status) (17.33 ± 11.79) assessed by BFMDRS. The percentage of improvement reached 70.6, 74.67, and 77.05%, respectively. At LFU, significant differences were found between the stimulation bi-OFF and uni-ON (11.08 ± 8.38 vs. 9 ± 8.52, *p* = 0.0191), and between the stimulation bi-OFF and bi-ON (11.08 ± 8.38 vs. 4.67 ± 4.72, *p* = 0.0164). Trends depicting a better improvement in stimulation bi-ON compared with uni-ON (4.67 ± 4.72 vs. 9 ± 8.52, *p* = 0.0538) were observed.

**Conclusion:**

Our results suggest that bilateral STN-DBS plus unilateral PVP may be an effective rescue procedure for patients with isolated dystonia who experienced suboptimal movement improvement following GPi-DBS. However, given the heterogeneity of patients and the small sample size, these findings should be interpreted with caution.

## Introduction

Isolated dystonia refers to a clinically and genetically heterogeneous group of movement disorders characterized by sustained and repetitive muscle contractions that often results in abnormal posturing and no other neurological abnormalities apart from tremor. The etiology of isolated dystonia can be classified as inherited, acquired, and idiopathic (Albanese et al., 2013). Most affected individuals experience educational withdrawal and social isolation, leading to a significant reduction in their quality of life. Current evidence indicates that the pathophysiology of isolated dystonia involves the dysfunction of the corticostriatal-thalamocortical circuit (Balint et al., 2018).

Deep brain stimulation (DBS) is a minimally invasive procedure for patients with dystonia, whether it is inherited or idiopathically isolated. And it is suitable for those resistant to systematic medications and botulinum toxin injections (Rodrigues et al., 2019). The globus pallidus internus (GPi) is a viable therapeutic target for DBS, and multiple studies have demonstrated that bilateral stimulation at GPi could effectively and safely improve the clinical symptoms and quality of life of patients with isolated dystonia (Kupsch et al., 2006; Meoni et al., 2017; Lin et al., 2019). The randomized controlled trial published in 2012 reported that GPi DBS could improve the dystonia severity of primary generalized or segmental dystonia by 47.9% at 6 months and 61.1% at 3 years (Volkmann et al., 2012). However, 10–20% of patients show improvement below 25–30% (Pauls et al., 2017). The therapeutic failure was either primary (i.e., patients who had never shown any response) or secondary (i.e., patients who experienced a loss of response after initial improvement) (Pauls et al., 2017). Additionally, the management of some patients remains difficult despite the exclusion of reversible and common complications, such as improper lead positioning, hardware issues, and inadequate programming.

One dual-target, crossover sham-controlled study (Schjerling et al., 2013) in 2013 examined 12 patients with dystonia (10 primary and 2 secondary) whose electrodes were implanted bilaterally in the GPi and subthalamic nucleus (STN). The report found that the Burke-Fahn-Marsden Dystonia Rating Scale (BFMDRS) movement scores were larger with four electrodes in service compared to bilateral stimulation at either target. These findings suggest that the simultaneous stimulation of GPi and STN may generate an additional value. However, the combination strategy means that the implantation of four electrodes and two sets of implanted pulse generators (IPGs), which will remarkably increase the economic burden and new trauma for additional IPG, is not applicable to a subset of patients.

Unilateral posteroventral pallidotomy (PVP) is an alternative surgical option for dystonia. Several studies demonstrate the comparable efficacies between PVP and GPi-DBS. Previous investigations have shown a much higher risk of employing a bilateral PVP than a unilateral procedure, although the efficacy of bilateral PVP in dystonia could reach a 50–90% alleviation in BFMDRS scores (Eltahawy et al., 2004; Horisawa et al., 2021). Recently, one study from Horisawa’s team reported the safety and efficacy of unilateral PVP for primary dystonia in all midline symptoms, including eyes, mouth, speech, swallow, and neck (Horisawa et al., 2021). Therefore, unilateral PVP remains a viable treatment option for patients with dystonia. In addition, it has a price advantage amounting to below 20% of the total cost for GPi (or STN) DBS in China. Therefore, PVP can be particularly appropriate for dystonia patients who cannot afford DBS therapy.

Two studies (Fonoff et al., 2012; Dec et al., 2014) indicated STN DBS increased further benefits for patients with dystonia who experienced partial improvement after the initial PVP, suggesting the synergistic effect of bilateral STN on PVP. Therefore, we hypothesized that STN-DBS plus unilateral PVP is an effective alternative for STN plus GPi-DBS after unsatisfactory GPi-DBS outcomes. Through the adoption of this surgical method, we acknowledge its cost-saving advantage, as the bilateral electrodes of STN can be connected to the previous IPG. Therefore, in this study, we aimed to investigate whether STN-DBS plus PVP is effective for patients with isolated dystonia who have undergone secondary failure of GPi-DBS.

## Materials and methods

### Patients

We recruited six patients at the Functional Neurosurgical Center of Shanghai Ruijin hospital from June 2018 to June 2020. The inclusion criteria were (i) diagnosis of isolated dystonia, including (1) dystonia and an otherwise normal neurological examination, (2) no history of other known etiologies of dystonia, (3) normal brain magnetic resonance imaging (MRI), (4) no family history of dystonia, (5) no previous exposure to medications possibly causing acquired dystonia, including levodopa and dopamine agonists, neuroleptics (dopamine receptor blocking drugs), anticonvulsants, and calcium channel blockers, and (6) no history of trauma, dementia (Mini-Mental State Examination score > 26) or other known metabolic and systemic causes; (ii) record of suboptimal bilateral GPi-DBS; (iii) adequate programming without obvious impact; and (iv) accurate location of the electrodes verified by the postoperative MRI (Supplementary Figure 1). A blinded independent expert rater assessed the correctness of the GPi lead placement.

All six patients experienced adequate programming strategies. In detail, if symptoms could not be controlled at 4.5 V or if stimulation-induced adverse effects hindered the further increase in voltage, reprogramming was performed using various procedures, including trying different combinations of large- and small-pulse widths and frequencies, the addition of other monopolar contacts, double monopolar stimulation, a bipolar stimulation mode, or interleaving stimulation. However, the results were either ineffective or included reports of adverse effects, encompassing dysarthria, increased muscle tone, gait disorders, paresthesia, and blurred vision. Table 1 presents the last set of stimulation parameters for GPi.

**TABLE 1:** Clinical characteristics and clinical outcomes for each patient.*^a^*

	Patient 1	Patient 2	Patient 3	Patient 4	Patient 5	Patient 6	Mean ± SD
Age at onset (year)	58	47	37	46	53	45	47.67 ± 7.20
Age at GPi DBS (year)	62	48	39	54	54	60	52.83 ± 8.40
Age at STN DBS plus PVP (year)	63	49	40	56	56	61	54.17 ± 8.47
Gender	M	M	M	F	M	F	
Duration (months)	50	12	25	101	17	300	84.17 ± 110.70
Body distribution	Segmental	Segmental	Focal	Focal	Segmental	Multifocal	NA
Affected regions	Eye, mouth, neck	Eye, neck	Neck	Neck	Eye, mouth	Mouth, limbs, neck	NA
Gene mutation	n.a.	None	None	None	None	n.a.	NA
Failed preoperative medication	Baclofen, diazepam	Trihexyphenidyl, tiapride, botulinum toxin	Baclofen, diazepam, trihexyphenidyl, botulinum toxin	Baclofen, diazepam, benzhexol hydrochloride, botulinum toxin	Botulinum toxin, diazepam	Diazepam, trihexyphenidyl	NA
Classification	Sporadic, isolated	Idiopathic sporadic, isolated	Idiopathic sporadic, isolated	Idiopathic sporadic, isolated	Idiopathic sporadic, isolated	Sporadic, isolated	NA
LFU after GPi DBS (months)	12	18	13	28	23	18	18.67 ± 6.06
LFU after STN DBS plus PVP (months)	24	19	15	13	12	13	16.00 ± 4.65
The last stimulation parameters for GPi (amplitude [V]/frequency [Hz]/pulse width [msec])	Lt: 3.55/160/70 case(+) 9(−); Rt: 3.75/160/70 case(+) 0(−)	Lt: 3.45/160/80 case(+) 8(−)9(−); Rt: 3.35/160/80 case(+) 0(−)1(−)	Lt: 3.0/140/110 case(+) 8(−); Rt: 3.5/140/90 case(+) 0(−)	Lt: 3.9/160/90 case(+) 8(−); Rt: 3.65/160/90 case(+) 0(−)	Lt: 3.75/160/70 case(+) 8(−)9(−); Rt: 2.95/160/90 case(+) 0(−)	Lt: 3.45/160/90 case(+) 8(−); Rt: 3.75/160/90 case(+) 0(−)	NA
Optimal stimulation Parameters for STN (amplitude [V]/frequency [Hz]/pulse width [msec])	Lt: 3.05/145/60 case(+) 10(−); Rt: 3.15/145/60 case(+) 2(−)	Lt: 2.85/130/60 case(+) 1(−); Rt: 2.35/130/60 case(+) 2(−)	Lt: 2.25/135/90 case(+) 10(−); Rt: 3.25/135/90 case(+) 2(−)	Lt: 2.95/145/60 case(+) 10(−); Rt: 2.25/145/60 case(+) 2(−)	Lt: 2.05/145/60 case(+) 3(−); Rt: 2.55/135/60 case(+) 3(−)	Lt: 1.7/170/90 case(+) 9(−); Rt: 2.5/170/90 case(+) 2(−)	NA

^a^None underwent gene test and found no mutation; n.a, did not do gene test; LFU, last follow-up; GPi, globus pallidus internus; STN, subthalamic nucleus; PVP, posteroventral pallidotomy; m/d scores, movement/disability scores; bi, biliteral; uni, unilateral; duration, duration before GPi DBS; NA, not applicable. Description statistics are shown with the mean ± standard deviation.

All six patients were unable to accept staged surgery due to superimposed surgical trauma or increased costs. Post-operative MRI also excluded the DBS lead malposition. Patients 2, 3, 4, and 5 completed the whole exome sequencing and no genetic mutations were found. *DYT1* and *DYT6* genes were routinely tested in patients with dystonia and the results of patients 1 and 6 were negative.

Patient 1 is a 63-year-old man who had a 6-year history of cervical and oromandibular dystonia, featuring difficulty in speech and swallowing before GPi DBS. The disorder began with torticollis, especially when he felt nervous. Three months later, the patient developed spontaneous mouth movements, inarticulacy, and resultant dysphagia. The patient repudiated the history of diabetes, hypertension, infectious diseases, alcohol addiction, smoking, and allergies. Treatment with baclofen and diazepam failed due to their intolerant side effects. From here on, his dysphagia further deteriorated. GPi DBS was performed at the age of 62. Considerable effects were observed in his neck after the operation. The improvements in his mouth and speech reached up to 46.15% in the first 6 months. However, the efficiency decreased later and eventually, recurrence emerged despite the repeated programming. Before the rescue procedures, the patient presented with cranial and cervical dystonia involving the oromandibular muscles, involuntary head rotated and tilted to the right, as well as dysarthria and dysphagia. He also complained of temporomandibular and cervical pain.

Patient 2 developed left torticollis and cervical pain without any known origin at the age of 47. These symptoms significantly improved after treatment with tiapride and baclofen. The medications were eventually suspended due to their side effects. A botulinum toxin injection was attempted 1 year later with considerable, but transient, benefits. Therefore, the patient underwent GPi DBS 2 years after the onset of symptoms. His cervical dystonia improved significantly after the stimulation, with a 66.67% reduction in BFMDRS scores; however, he began to experience foreign body sensations in his eyes, photophobia, and blurred vision 6 months post-surgery. Soon, his eyes started to blink involuntarily, and the frequency of blinking gradually increased. Additionally, the previously relieved cervical dystonia got worse.

Patient 3 suffered from left torticollis at age 37 for an unknown reason. Initially, the twisting was intermittent, occurring 2–3 times a day. The frequency increased within 2 months and was accompanied by neck pain. He tried treatment with baclofen, diazepam, and trihexyphenidyl successively without evident amelioration. At the age of 38, the patient had a botulinum toxin injection, resulting in partial alleviation. However, after three treatments, the efficacy gradually disappeared. He underwent GPi-DBS 2 years after the onset of symptoms (39 years old), and he reported a 50% decrease in BFMDRS scores. The patient was unsatisfied with the effects of this procedure and his symptoms also started to fluctuate. Upon examination prior to the second operation, the patient presented with left torticollis, neck pain, and cervical stiffness.

Patient 4 suffered from neck pain without a known reason. The patient’s cervical tilting angle to the right gradually reached 160° at the age of 46. In the beginning, the symptoms occurred occasionally and were relieved by the sensory trick. Two years after onset, the symptoms aggravated with an upregulated frequency and persistent pain. Baclofen, diazepam, and benzhexol hydrochloride were prescribed and a botulinum toxin injection was given. However, the torticollis further deteriorated with the head becoming fixed to the left. There is no history of hypertension, diabetes, infectious diseases, smoking, alcohol addiction, and allergies in this patient. She received GPi-DBS at the age of 54 and her maximum improvement percentage amounted to 83.3%. However, the pre-operative symptoms reemerged during the 11-month follow-up and a novel symptom of shoulder muscle tension appeared.

Patient 5 suffered from progressive blepharospasm first noted at 53 years old, without any related history and evidence of a psychogenic disorder. It was followed by uncontrolled jerking in the inferior face and severe tongue spasms, resulting in inarticulate speech. Scans from brain magnetic resonance imaging (MRI) were normal. Treatment with botulinum toxin, diazepam, and trihexyphenidyl produced little benefit. He accepted GPi-DBS 1 year later. The symptoms in his eyes and mouth were relieved during the first 6 months postoperatively, with a 75% reduction in BFMDRS scores. However, the symptoms recurred and intensified afterward, with durative blepharospasm, constant mouth movements, and a low speaking tone. Upon preoperative assessment, the patient showed severe blepharospasm and oromandibular dystonia.

Patient 6 was admitted to our hospital when she was 60 years old, with a chief complaint of involuntary movement in the mouth and upper limbs. The abnormality was intensified when performing tasks that require fine motor skills, such as writing. There is no family history of any movement disorder and no record of any relevant medication intake. The general practitioner prescribed haloperidol and clonazepam, which brought about transient improvement and eventually followed by deterioration. Before GPi-DBS, there were sustained involuntary actions in both arms and the speech was slurred. GPi-DBS was performed after a complete evaluation. The symptoms took a favorable turn in the first 6 months, with a 54.54% improvement. However, the efficiency decreased 8 months later, and repeated programming could not alleviate the symptoms. Before the alternative surgery was performed, the patient displayed severe involuntary movement in the arms, shoulders, neck, and mouth.

### Clinical evaluations

Clinical evaluations were performed at baseline and at 1 month, 6 months, and LFU (12–24 months; see Table 1) postoperatively. All patients were assessed with the movement and disability subscales of the BFMDRS and SF-36. The LFU estimation was conducted with the STN bilateral OFF, unilateral ON (opposite side of the PVP), and bilateral ON states. The patients were first examined under STN bilateral ON, and then they were evaluated 12 h after STN unilateral ON. All six patients completed this step. Then, the other side of STN was switched-off for another 12 h. However, patients 2 and 5 were unable to tolerate the abrupt worsening of the dystonia (i.e., could not open their eyes) within 30 min after the bilateral switch-off. Therefore, clinical evaluation was performed immediately, and DBS was reinitiated within 30 min upon request of the patients. The other four patients completed the whole process, although symptom deterioration occurred within the first 30 min after STN was bilaterally switched off. All subjects confirmed reaching their original DBS clinical effect within 3 days of rebooting the bilateral stimulation. A trained rater who was blinded to the group status scored each follow-up according to standardized criteria. A specialist who was not blinded saw the patients regularly in the outpatient clinic to adjust the DBS parameters based on their clinical responses.

### Surgical procedures

**FIGURE 1:**
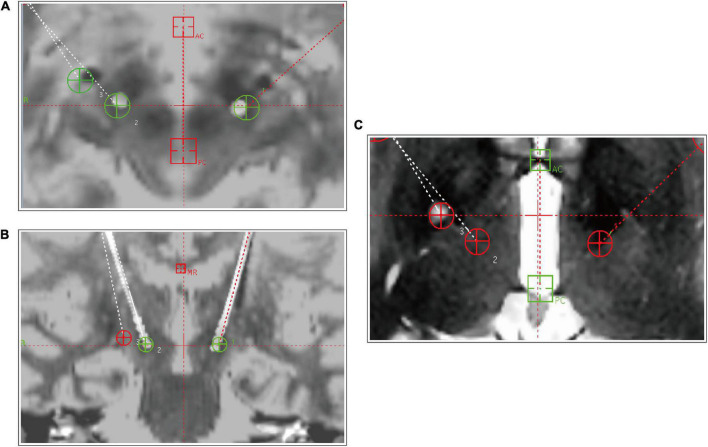
Postoperative MR images of patient 5, demonstrating positions of the implanted electrodes in the bilateral STN **(A,B)** and unilateral pallidotomy. The two red orthogonal lines refer to the Cartesian coordinate system in each view, whereas the diagonal lines, with or without green circles, represent the trajectories of the implanted leads. In the center of each view, the two green circles (named 1 and 2) in **(A,B)** show the planned targets, and the one red circle (named 3) in **(C)** shows the position of pallidotomy. AC, anterior commissure; MR, midline reference; PC, posterior commissure.

A Leksell stereotactic frame (Elekta, Stockholm, Sweden) was mounted on the patient’s head under local anesthesia prior to obtaining a computed tomography (CT) scan. The fusion image was obtained by merging the images from CT and MRI (1.5 T, General Electric) using the Surgiplan software (Elekta, Stockholm, Sweden) for GPi and STN targeting, as previously described (Lin et al., 2019). Under local anesthesia, the previously implanted GPi-DBS lead was pulled out and PVP was performed. The GPi was located 2–4 mm anterior to the anterior commissure–posterior commissure (AC-PC) line midpoint, 18–22 mm lateral to the AC-PC line, and 2–4 mm below the AC-PC line. A radiofrequency electrode (Radionics) with a 2-mm diameter radiofrequency probe and a 2-mm exposed tip was used for impedance measurement. The tip of the electrode was heated to 70–80°C for 60 s. The length of the lesion was about 5 mm. New Quadripolar DBS electrodes (model 3387, Medtronic) were then implanted into the STN and connected to the previously implanted extension wire and IPG (37612 RC or 37603 SC, Medtronic) under general anesthesia. Postoperative MRI and CT confirmed the precision of PVP and electrode placement (Figure 1) and the targeted and actual (post-op imaging-derived) anterior commissure AC-PC coordinates of the STN leads and PVP are listed in Supplementary Table 1.

The surgery site for unilateral PVP was decided based on the dystonia distribution. Generally, the contralateral hemisphere to the most affected side by dystonia was chosen as the surgical site. For patients 1, 2, 3, and 4 who all exhibited asymmetrical cervical dystonia, laterocollis. Therefore, the contralateral side to the direction of neck tilting was chosen as the surgical side, which was consistent with another article we reported (Lai et al., 2020). For example, patient 3 presented with left laterocollis and underwent right PVP. Patients 1, 2, and 4 presented with right laterocollis and underwent left PVP. For patient 5, he presented symmetrical midline symptoms. For him, the right PVP was chosen. Unilateral PVP was reported to significantly improved all midline BFMDRS subitems (eyes, mouth, speech/swallow, neck, and trunk) (Horisawa et al., 2021). However, studies have shown that left PVP produced more impairment in verbal fluency than right PVP (Crowe et al., 1998; Junqué et al., 1999). Therefore, for patients only presenting with symmetrical symptoms, the right PVP is preferred. For patient 6, she presented more severe right upper limb symptoms, and left PVP was chosen for her.

### Postoperative stimulation parameters and statistical analysis

The patients were discharged from the hospital 1 week after surgery, and stimulation parameters were adjusted in an outpatient setting according to the patient’s clinical status at each follow-up postoperatively. All statistical analyses were performed using Graphpad prism 8. The differences in DBS efficacy after each follow-up are analyzed by parametric tests (Student paired-sample *t*-tests) or non-parametric models (paired-sample Wilcoxon signed-rank tests). A *p*-value of < 0.05 was considered statistically significant.

## Results

### Demographics and clinical data

Table 1 presents the clinical characteristics and preoperative scores of each patient (two females and four males). The age of patients undergoing surgery ranges from 40 to 63 years old.

### Outcomes of dystonia

**FIGURE 2:**
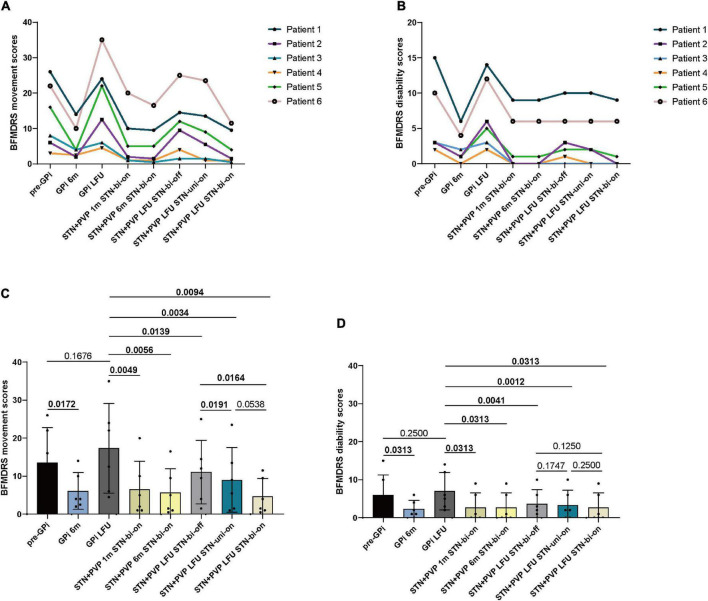
Individual BFMDRS movement **(A)** and disability scores **(B)** before bilateral GPi DBS surgery (pre-GPi), at 6 months (GPi 6m) and the last follow-up (LFU) after GPi DBS (GPi LFU), and at 1 month (STN + PVP 1m STN-bi-on), 6 months (STN + PVP 6m STN-bi-on), and LFU post-bilateral STN plus unilateral PVP surgery. The LFU post-bilateral STN plus unilateral PVP was evaluated at three conditions: STN bilateral OFF (STN + PVP LFU STN-bi-off), STN unilateral ON (STN + PVP LFU STN-uni-on), and STN bilateral ON (STN + PVP LFU STN-bi-on). **(C)** Mean BFMDRS movement scores and **(D)** disability scores at each follow-up. LFU, last follow-up; m, month; Pre, pre-operation; PVP, posteroventral pallidotomy; bi, bilateral; uni, unilateral. *P*-values for comparisons between each follow-up are analyzed by parametric tests (Student paired-sample *t*-tests) or non-parametric models (paired-sample Wilcoxon signed-rank tests). A *p*-value of < 0.05 was considered statistically significant.

Based on total movement BMFDRS scores, significant amelioration was achieved at 1-month (6.5 ± 7.45; *p* = 0.0049), 6-month (5.67 ± 6.3; *p* = 0.0056), and at LFU (4.67 ± 4.72; p = 0.0094) follow-up compared with the baseline (LFU of GPi DBS with on status) (17.33 ± 11.79). The percentage of improvement reached 70.6, 74.67, and 77.05%, respectively (Table 2 and Figure 2). At LFU, a significant difference was found between stimulation bi-OFF and uni-ON (11.08 ± 8.38 vs. 9 ± 8.52, *p* = 0.0191), as well as between stimulation bi-OFF and bi-ON (11.08 ± 8.38 vs. 4.67 ± 4.72, *p* = 0.0164).

**TABLE 2:** Effect of treatment on BFMDRS movement and disability scales after surgery.^a^

Variable	Patient 1 (m/d)	Patient 2 (m/d)	Patient 3 (m/d)	Patient 4 (m/d)	Patient 5 (m/d)	Patient 6 (m/d)	Movement scores (mean ± SD)	Disability scores (mean ± SD)	Mean improvement, % (movement scores)	Mean improvement, % (disability scores)
pre-GPi	26/15	6/3	8/3	3/2	16/3	22/10	13.5 ± 9.29	6 ± 5.29	/	/
GPi 6m	14/6	2/1	4/2	0.5/0	4/1	10/4	6.08 ± 4.82	2.33 ± 2.25	62.61	63.82
GPi LFU	24/14	12.5/6	6/3	4/2	22/5	35/12	17.33 ± 11.79	7 ± 4.9	-34.26	-30
STN + PVP 1m	10/9	2/0	1/0	1/0	5/1	20/6	6.5 ± 7.45	2.67 ± 3.88	70.6	77.62
STN + PVP 6m	9.5/9	1.5/0	0.5/0	1/0	5/1	16.5/6	5.67 ± 6.3	2.67 ± 3.88	74.67	77.62
STN + PVP LFU STN-bi-off	14.5/10	9.5/3	1.5/0	4/1	12/2	25/6	11.08 ± 8.38	3.67 ± 3.72	42.59	56.43
STN + PVP LFU STN-uni-on	13.5/10	5.5/2	1.5/0	1/0	9/2	23.5/6	9 ± 8.52	3.33 ± 3.93	60.44	67.54
STN + PVP LFU STN-bi-on	9.5/9	1.5/0	0.5/0	1/0	4/1	11.5/6	4.67 ± 4.72	2.67 ± 3.88	77.05	77.62

***P***-**value^b^**										

Variable	GPi 6m vs. pre-GPi	GPi LFU vs. pre-GPi	GPi LFU vs. STN + PVP 1m	GPi LFU vs. STN + PVP 6m	GPi LFU vs. STN + PVP 12m bi STN-off	GPi LFU vs. STN + PVP 12m uni STN-on	GPi LFU vs. STN + PVP 12m bi STN-on	STN + PVP 12m STN-bi-off vs. 12m STN-uni-on	STN + PVP 12m STN-uni-on vs. 12m STN-bi-on	STN + PVP 12m STN-bi-off vs. 12m STN-bi-on
Movement scores	**0.0172**	0.1676	**0.0049**	**0.0056**	**0.0139**	**0.0034**	**0.0094**	**0.0191**	0.0538	**0.0164**
Disability scores	**0.0313**	0.25	**0.0313**	**0.0313**	**0.0041**	**0.0012**	**0.0313**	0.1747	0.25	0.125

^a^Pre, preoperative. BFMDRS scores in each patient are shown in (m/d). m/d, BFMDRS movement scores/BFMDRS disability scores. Description statistics are shown with the mean ± standard deviation; % improvement in the post-GPi = BFMDRS score (baseline—6 months or LFU)/baseline; % improvement in the post-PVP + STN = BFMDRS score (GPi LFU-each follow-up after PVP + STN)/GPi LFU.

^b^P-value for comparisons between each follow-up as analyzed by parametric tests (Student paired-sample t-tests) or non-parametric models (paired-sample Wilcoxon signed-rank tests). The bold values refers to the p values below 0.05.

The total disability BFMDRS scores reduced significantly at 1-month (2.67 ± 3.88; *p* = 0.0313), 6-month (2.67 ± 3.88, *p* = 0.0313), and 12-month follow-up (2.67 ± 3.88; *p* = 0.0313) compared with baseline (7 ± 4.9), with an improvement of 77.62%, respectively (Table 2).

### Assessment of quality of life

STN plus STN DBS remarkably upregulated the quality of life evaluated by SF-36, 1, 6, and 12 months postoperatively (Table 3). Noticeable elevation was discovered in every subscale of SF-36 except for role physical, especially in general health and mental health, aspects reaching a *p*-value lower than 0.01.

**TABLE 3:** Health-related quality of life data as a function of STN + PVP before surgery and at 1, 6, and LFU months after surgery.^a^

	Score: Mean ± SD						*P*-value *^b^*				
		
SF36 subscale	Pre_GPi	GPi_6m	GPi LFU	STN + PVP 1m STN_bi_on	STN + PVP 6m STN_bi_on	STN + PVP 12m STN_bi_on	GPi_6m vs. Pre_GPi	GPi_LFU vs. Pre_GPi	STN + PVP 1m vs. GPi LFU	STN + PVP 6m vs. GPi LFU	STN + PVP 12m vs. GPi LFU
General health	22 ± 6.8	58 ± 13	20 ± 6.3	47 ± 9.3	68 ± 5.2	69 ± 6.6	0.0006	0.6383	0.0001	<0.0001	<0.0001
Physical function	36 ± 21	77 ± 19	29 ± 22	78 ± 20	82 ± 25	90 ± 22	0.0313	0.5	0.0003	0.0313	0.0313
Role physical	21 ± 40	50 ± 55	21 ± 40	50 ± 55	67 ± 52	67 ± 52	0.5	/	0.5	0.25	0.25
Role emotional	11 ± 17	44 ± 11	11 ± 17	50 ± 28	83 ± 18	89 ± 17	0.0625	/	0.0625	0.0313	0.0313
Social functional	21 ± 19	46 ± 10	21 ± 10	54 ± 19	79 ± 19	79 ± 19	0.0625	>0.9999	0.0313	0.0313	0.0313
Body pain	50 ± 10	70 ± 6	50 ± 12	72 ± 8	77 ± 5.2	77 ± 5.2	0.0625	>0.9999	0.0005	0.0313	0.0313
Vitality	37 ± 12	58 ± 8	34 ± 8	58 ± 7.5	73 ± 9.4	77 ± 11	0.0026	0.5177	<0.0001	0.0002	0.0313
Mental health	35 ± 4.1	62 ± 21	31 ± 13	57 ± 17	65 ± 18	69 ± 19	0.0625	0.625	0.0019	0.0012	0.0012

^a^LFU, last follow-up.

Values are expressed as the mean ± standard deviation. Scores range from 0 to 100, and an increase in score indicates improvement.

^b^p-value for every subscale comparison between 1 month and pre-operation, 6 months and 1 month, and LFU and 6 months in each group.

### Adverse events

Overall, the surgical procedures were well-tolerated in this population. There were no hardware-related side effects, infections, intracranial hemorrhages, or extension or lead fractures from DBS implantation during the follow-up period. Although patient 2 experienced dysarthria due to stimulation intensities above the therapeutic threshold, it was eliminated immediately after reprogramming. Stimulation-induced paresthesia took place in all six patients but vanished after adjusting the stimulation parameters. Common adverse events associated with STN-DBS in patients with Parkinson’s disease, including fatigue and dyskinesia, were not observed in any of our patients.

## Discussion

Here, STN-DBS plus unilateral PVP significantly improved overall movement and disability BFMDRS scores by 77.05 and 77.62%, respectively, at the final follow-up (mean 16.00 ± 4.65 months) in patients with previously failed GPi-DBS.

There were few reports available considering rescue strategies for suboptimal DBS in dystonia. Thus, for the literature review, we used the search terms “dystonia” and “thalamotomy” or “pallidotomy” or “subthalamic nucleus” or “globus pallidus internus” or “lesional surgery” in combination with “failed,” “previously undergone,” “prior,” “suboptimal,” or “rescue” in PUBMED and EMBASE databases. All articles in English published before 10 April 2022 were included. The full text was checked to select the studies investigating practice for unsatisfied response to GPi-DBS. Ultimately, four original studies including case reports were identified for further discussion (Table 4). Ellis et al. (2008) did a case series with four patients receiving lead replacement (average distance of adjustment: 6.7 mm, bilateral or unilateral) after a less satisfying response to bilateral GPi-DBS. Two patients had their neck dystonia greatly relieved while one had benefits for motor symptoms and the other had mild recovery in speech and swallowing. Similarly, Oyama et al. (2011) reported the inconformity of lead position in one patient whose left GPi lead was 2.4 mm more anterior than the right one indicated by neuroimaging. Thus, the replacement was implemented followed by achieving the desired effect. Aragão et al. (2021) reported a patient with refractory Meige syndrome who was initially stimulated at GPi and achieved satisfactory alleviation after shifting the target to STN. Likewise, Oyama et al. (2011) reported a patient with dystonia received noticeable symptomatic relief after bilateral STN-DBS, which was the rescue procedure 2 years after the unsatisfying bilateral GPi-DBS.

**TABLE 4:** Reports of rescue procedures after failed DBS or lesion surgery in patients with dystonia.*^a^*

Author, year		Aragão et al., 2021	Ellis et al., 2008				Oyama et al., 2011		Blomstedt et al., 2016
Diagnosis		Meige syndrome	D	D	D	D	CD	TD	D
Age at onset (years)		65	66	40	8	43	32	25	10
Disease duration*^a^* (months)		228	7	5	3	12	13	8	732
Last follow-up (months)		24	12	24	6	6	17	15	12
Previous surgery		bi GPi DBS	bi GPi DBS	bi GPi DBS	bi GPi DBS	bi GPi DBS	bi GPi DBS	bi GPi DBS	bi GPI DBS (hardware-infection).
Rescue procedure		bi STN DBS	Replace leads-bi	Replace lead-uni	Replace leads-bi	Replace leads-uni	GPi DBS (L)	Bi STN DBS	Uni Pdt
UDRS	Baseline	NA	NA	NA	NA	NA	11	28	NA
	Before rescue	NA	22	NA	50	6	6	24	NA
	Post rescue	NA	18	NA	46	4	4	8	NA
	Improvement	NA	45.4%	NA	8%	33.3%	33.3%	66.7%	NA
BFMDRS	Baseline	NA	NA	NA	NA	NA	NA	NA	39
	Before rescue	17	NA	NA	NA	NA	NA	NA	2.5
	Post rescue	1.5	NA	NA	NA	NA	NA	NA	5
	Improvement	92.10%	NA	NA	NA	NA	NA	NA	-50%

^a^bi, Bilateral; uni, unilateral; D, Dystonia; CD, cervical dystonia; TD, Torsion dystonia; Pdt, Pallidotomy; UDRS, Unified Dystonia Rating Scale; NA, Not available; Duration, between onset and rescue procedure; improvement, before rescue and last follow-up.

Multiple factors could contribute to insufficient outcomes after GPi-DBS in isolated dystonia. Pauls et al. (2017) analyzed 22 isolated dystonia cases with Gpi-DBS failure and found lead displacement and inappropriate stimulation are the most common causes and thus should be excluded first. In our study, we ruled out these possibilities by verifying lead placement with postoperative MRI (Supplementary Figure 1) and sufficient programming. And the considerable improvement generated in the first 6 months (46.15–83.33%) further confirmed the initially accurate placement and suitable stimulating parameters.

Body distribution of dystonia may affect long-term outcomes. In our cohort, the areas involved were mainly cranial-cervical and cranial-facial. It was reported that cranial and cervical dystonia exhibit variant outcomes after GPi-DBS. Limotai et al. (2011) reported a remarkable variation of improvement among six patients with cranio-facial and cranio-cervical dystonia (reduction percentage of 16.6–100% indicated by BFMDRS scores) 12 months after GPi DBS, with two of them having less than 20% amelioration. The investigation from Sensi et al. (2009) showed that the improvement percentage of BFMDRS ranged from 30 to 82% in the long run for patients with segmental dystonia treated with GPi DBS. Martinez-Torres et al. (2009) reported that GPi DBS improved trunk and oropharyngeal dystonia but the benefit was absent for blepharospasm in isolated dystonia. Larger and longer prospective studies with blinded evaluation are needed to explore whether the regions involved are indicators for response to GPi-DBS and the underlying mechanisms.

Another reason worth considering is habituation. The term “habituation,” previously known as “tolerance,” is referred to as the vanishing of DBS efficacy despite reprogramming that could not be explained by loss of micro-lesional implant effect or disease progression (Fasano and Helmich, 2019; Peters and Tisch, 2021). It is mostly reported in cases of essential tremor cases, but the phenomenon has also been described in dystonia patients receiving GPi DBS (Shah and Jimenez Shahed, 2014). Currently, the underlying mechanism remains unclear. It is well-established that the dysfunction of the cortico-basal ganglia-thalamo-cortical circuit is a crucial contributor to dystonia (Vitek, 2002). Previous studies have shown that GPi-DBS could normalize excessive cortical plasticity and is one of the fundamental factors for its effect (Tisch et al., 2007; Ruge et al., 2011a; Barow et al., 2014). However, it has been suggested that habituation may also be generated from neural reorganization (Dostrovsky and Lozano, 2002; Ruge et al., 2011a; Peters and Tisch, 2021). And theoretically, it should be noted that STN DBS may also induce habituation. Nevertheless, in our cohort, all six patients relapsed within 1 year after GPi DBS; in contrast, no recurrence was reported before our last follow-up (12–24 months). A long-term follow-up is still needed.

Disease progression can also contribute to the decline of DBS efficacy. However, it is difficult to distinguish natural disease progression from habituation (Ruge et al., 2011a; Peters and Tisch, 2021). The emergence of new symptoms may be an indicator of disease deterioration. Therefore, in our study, the novel blepharospasm by patient 2 and the newly emerged shoulder muscle tension shown by patient 5 are possibly derived from disease deterioration.

In our study, the efficiency of PVP alone may be reflected by the status at bilateral STN OFF. While this conclusion must be considered with caution because it is possible that effects generated by STN DBS may not be washed out completely, it remains interesting that the unilateral PVP was highly effective and could rescue the failed bilateral GPi DBS. This may be related to the different mechanisms of action between these two procedures. Since GPi consists of gamma-aminobutyric-acid mediated inhibitory neurons, DBS at this location will lead to neural depolarization and subsequently suppresses abnormally enhanced synchronized oscillatory activity within the motor cortico-basal ganglia network in dystonia (Dostrovsky and Lozano, 2002; Ni et al., 2018). As for PVP, it may correct the irregular neuronal firing in the network by destroying the afferent and/or efferent circuitries (Lozano et al., 1997; Vitek et al., 1999). In addition, as mentioned before, DBS may probably generate habituation. Dystonic disorders are commonly characterized by strengthened plasticity and decreased inhibition in the motor cortex (Ridding et al., 1995; Quartarone et al., 2003). The investigation from Ruge and his co-workers suggested that these two parameters were normalized in the primary dystonia at 3- and 6-month follow-up (Ruge et al., 2011b) but showed distinct patterns from healthy controls in the long run (Ruge et al., 2011a). In our cohort, time points when the decay of established stimulation benefits took place were more than 6 months after implantation. Hence, though Ruge’s observations might be influenced by the bias of the small sample size and different genetic backgrounds, non-beneficial impacts from continuous stimulation may exist and may partly contribute to the unsatisfied response. Moreover, thalamotomy was indicated effective for failed thalamic DBS (Bahgat et al., 2013; Peters and Tisch, 2021), suggesting a possible disparity of effects between ablation and DBS as well.

The synergistic effect of unilateral PVP plus STN DBS was observed when comparing the benefits with that of bilateral-off, unilateral-on, and bilateral-on status of STN DBS at the last follow-up (Figure 2). There is growing evidence that dystonia is the reflection of multi-level network dysfunction (Jinnah et al., 2017). Therefore, stimulating different sites of the circuit spontaneously may generate combinational effects. Schjerling and his co-workers suggested double stimulation at GPi and STN was more effective than stimulating either target alone in dystonia (Schjerling et al., 2013). Two teams (Fonoff et al., 2012; Dec et al., 2014) reported STN DBS could generate further alleviation in patients with dystonia after partial improvement yielded from initial PVP, suggesting the collaborative effect of these strategies. Moreover, Horisawa et al. (2019) performed lesions at contralateral Forel’s field H1, the efferent fibers from the Gpi to the thalamus, on 11 patients with dystonia who had undergone unilateral PVP. They proposed the significant improvement observed derived from the congenerous effects of the combined surgeries. It is worth mentioning that Forel’s field H1 is located close to the dorsal border of the STN, which is the preferred target of STN in dystonia (Cao et al., 2013; Ostrem et al., 2017). Thus, the combined effect of unilateral PVP plus STN DBS in our study may have a similar mechanism to the unilateral PVP plus contralateral campotomy.

There are few reports exploring the washout time of STN DBS in dystonia. Miocinovic et al. (2018) performed a 90-min for DBS washout and worsen dystonia was observed, but the performance would not drop back to that at baseline. Wagle Shukla et al. (2018) adopted 4–8 h for washout of STN stimulation and a significant worsening of dystonic symptoms was observed. In our study, even though a 12-h washout was used and significant upregulation of BFMDRS scores was observed, insufficient washout could not be excluded. Further exploration of washout time on DBS for dystonia is necessary.

Patient 6 showed upper limb torsion, which was less common in isolated dystonia. After excluding neuropathy abnormalities, such as neurodegeneration, acquired impairment (like intracerebral lesions), metabolism, or other systemic factors, she was finally diagnosed with isolated dystonia (idiopathic or genetic etiology) according to the consensus in 2013 (Albanese et al., 2013). This diagnosis was supported by Bettina Balint and her team who reviewed the cases of idiopathic or genetic isolated dystonia and found that upper limb involvement was a typical clinical manifestation of monogenic dystonia (Balint et al., 2018).

The current report described the effectiveness and safety of bilateral STN-DBS plus unilateral PVP in six patients with isolated dystonia who had previously undergone unsatisfactory GPi-DBS. This rescue procedure was selected for the following reasons: First, it is cost-saving without an additional IPG, compared with bilateral stimulation at both GPi and STN. Second, it is also suitable for patients who prefer not undergoing staged surgery or having two implanted IPGs. In these cases, whether they have financial concerns or not, STN plus PVP is a viable alternative option for them to choose.

There are several limitations to this study. First, the sample size is small and the background is relatively heterogenous since two subjects declined whole exome sequencing. This may lead to a deviation in our results because the response to GPi or STN-DBS may vary depending on certain genetic backgrounds (Aravamuthan et al., 2017). Second, patients 2 and 5 could not tolerate a bilateral STN-off. Therefore, upon request, we switched on STN in advance, which might introduce some bias into the results. Third, owing to the worsening of dystonia in the stimulation “off” state, blinding the participants to stimulation status was not possible. Fourth, the time period for DBS OFF was relatively short and may not achieve a complete washout thus influencing the evaluation of PVP’s effect. Fifth, our study could not conclude whether the efficiency of PVP plus STN-DBS is better than the effect achieved by STN-DBS alone due to the persistent effect of PVP. It is more rigorous to conduct a staged surgery that the bilateral STN DBS is first applied and PVP can be considered according to STN-DBS’s effect. Our strategy is suitable for patients who are unwilling to undergo two surgeries with superimposed surgical trauma. Future studies should enroll eligible patients to address this issue.

## Conclusion

This study confirmed the significant improvement in BFMDRS motor scores (77.05% reduction) during the 16-month follow-up after bilateral STN-DBS plus unilateral PVP in patients with isolated dystonia who experienced secondary failure following GPi-DBS. The bilateral STN-DBS plus unilateral PVP may be an alternative rescue procedure for isolated dystonia. Larger and longer prospective studies with blinded evaluation are needed to elucidate the effect of bilateral STN-DBS plus unilateral PVP on dystonia.

## Data availability statement

The raw data supporting the conclusions of this article will be made available by the authors, without undue reservation.

## Ethics statement

The studies involving human participants were reviewed and approved by the Ruijin Hospital Ethics Committee of the Shanghai Jiao Tong University School of Medicine. The patients/participants provided their written informed consent to participate in this study. Written informed consent was obtained from the individual(s) for the publication of any potentially identifiable images or data included in this article.

## Author contributions

DL, YW, SL, and BS contributed to conception and design. SL, SG, YW, TW, LW, CZ, and YS conducted the acquisition of data. SL, YW, LW, SG, and HL analyzed and interpreted the data. SL, LW, and HL drafted the article. DL, SL, YW, LW, YS, SG, and CZ critically revised the article. DL, SL, YW, CZ, LW, SG, and TW gave administrative, technical, and material support. DL, SL, YW, and LW offered study supervision. All authors reviewed the manuscript for submission and YW approved the final version of the manuscript on behalf of all authors.

## Abbreviations

GPi: Globus pallidus internus
DBS: deep brain stimulation
STN: subthalamic nucleus
PVP: posteroventral pallidotomy
LFU: last follow-up
BFMDRS: Burke-Fahn-Marsden Dystonia Rating Scale
IPGs: implanted pulse generators
MRI: magnetic resonance imaging
VTA: tissue activated
CT: computed tomography
SCP: superior cerebellar peduncle
DN: dentate nucleus.
