# Supplementary material for: Rescue procedure for isolated dystonia after the secondary failure of globus pallidus internus deep brain stimulation
Source: Front Neurosci. 2022 Aug 18;16:924617. doi: 10.3389/fnins.2022.924617 (PMC9434021; doi:10.3389/fnins.2022.924617)
Supplement: Supplementary file 1 [file Data_Sheet_1.docx]

Supplementary Material

Supplementary Table 1. The x-y-z coordinates of actual and target contacts ^1^

| No. | Targets | R-x | | R-y | R-z | L-x | | L-y | | L-z |
| --- | --- | --- | --- | --- | --- | --- | --- | --- | --- | --- |
| Patient 1 | STN | -15.1(-14.5) | | 2.9(3.1) | 5.2(4.4) | 16.4(17) | | 3.2(3.5) | | 4.9(5.2) |
|  | GPi (L) |  | |  |  | 21.7(21.5) | | 2.0(2.0) | | 4(4.1) |
| Patient 2 | STN | -12.1(-12) | | 2.7(2.1) | 3.8(4.0) | 13(13.5) | | 3.2(2.7) | | 4.5(5.0) |
|  | GPi (L) |  | |  |  | 19.2(19.1) | | 2.8(2.8) | | 5.7(4.5) |
| Patient 3 | STN | -13.7(-14.2) | | 3.0(3.5) | 6.0(6.5) | 13.3(12.9) | | 3.5(4.2) | | 4.9(5.2) |
|  | G®(R) | -21.8(-21.6) | | 3.9(4.1) | 6.4(6.2) |  | |  | |  |
| Patient 4 | STN | -12.0 | | 4.3 | 5.6(4.6) | 13(13.5) | | 4.3(3.3) | | 5.2(5.5) |
|  | GPi (L) |  | |  |  | 17.6(17.4) | | 2.6(2.7) | | 4.8(4.8) |
| ®Pi (R) | -20.4(-20.1) | | 1.7(2.0) | 4(4.4) |  | |  |  |  |  |
| Patient 6 | STN | -10.9(-10.3) | | 4.4(4.9) | 4.8(5.8) | 11.8(12) | | 3.0(2.8) | | 5.0(6.0) |
|  | GPi (L) |  | |  |  | 17.7(17.7) | | 2.5(2.6) | | 4.7(4.6) |

^a^ GPi, globus pallidus internus; STN, subthalamic nucleus; x-y-z coordinate system is, the x-axis would go from front to back, the y-axis would go from left to right, and the z-axis would go from up to down. Mid-point (MC) of the AC–PC line is x=0, y=0, z=0. The x, y, and z-coordinates of a point are the distances from MC point on the yz-plane, xz-plane, and xy-plane, respectively; R, right; L, left. The numbers in the brackets are actual coordinates, and the numbers outside the brackets are target coordinates.

| Movement scores | **0.0172** | 0.1676 | **0.0049** | **0.0056** | **0.0139** | **0.0034** | **0.0094** | **0.0191** | 0.0538 | **0.0164** |
| --- | --- | --- | --- | --- | --- | --- | --- | --- | --- | --- |
| Disability scores | **0.0313** | 0.25 | **0.0313** | **0.0313** | **0.0041** | **0.0012** | **0.0313** | 0.1747 | 0.25 | 0.125 |

^1^ Pre, preoperative. BFMDRS scores in each patient are shown in (m/d). m/d, BFMDRS movement scores/BFMDRS disability scores. Description statistics are shown with the mean ± standard deviation; % improvement in post-GPi=BFMDRS score (baseline-6m or LFU)/baseline; % improvement in post-PVP+STN=BFMDRS score (GPi LFU-each follow up after PVP+STN)/GPi LFU;

^2^ P-value for comparisons between each follow-up as analyzed by parametric tests (Student paired-sample t-tests) or nonparametric models (paired-sample Wilcoxon signed-rank tests).

Supplementary Table 2. Raw data of BFMDRS scores

| Patient No | Follow-up | Total score | Movement scale | | | | | | | | | |  | Disability scale | | | | | | | |
| --- | --- | --- | --- | --- | --- | --- | --- | --- | --- | --- | --- | --- | --- | --- | --- | --- | --- | --- | --- | --- | --- |
|  |  |  | eyes | mouth | speech/swallowing | neck | R-arm | L-arm | trunk | R-leg | L-leg | total1 |  | speech | hantwriting | feeding | eating/swallowing | hygiene | dressing | walking | total2 |
| Patient1 | Pre_op | 41 | 0 | 6 | 12 | 8 | 0 | 0 | 0 | 0 | 0 | 26 |  | 1 | 0 | 4 | 3 | 3 | 1 | 3 | 15 |
|  | GPi 6m | 20 | 0 | 4 | 6 | 4 | 0 | 0 | 0 | 0 | 0 | 14 |  | 1 | 0 | 2 | 1 | 1 | 1 | 0 | 6 |
|  | GPi LFU | 38 | 0 | 6 | 12 | 6 | 0 | 0 | 0 | 0 | 0 | 24 |  | 1 | 0 | 4 | 3 | 2 | 1 | 3 | 14 |
|  | STN+PVP 1m STN_bi_on | 19 | 0 | 3 | 6 | 1 | 0 | 0 | 0 | 0 | 0 | 10 |  | 2 | 0 | 2 | 2 | 1 | 1 | 1 | 9 |
|  | STN+PVP 6m STN_bi_on | 18.5 | 0 | 3 | 6 | 0.5 | 0 | 0 | 0 | 0 | 0 | 9.5 |  | 2 | 0 | 2 | 2 | 1 | 1 | 1 | 9 |
|  | STN+PVP LFU STN_bi_off | 24.5 | 0 | 4.5 | 8 | 2 | 0 | 0 | 0 | 0 | 0 | 14.5 |  | 2 | 0 | 2 | 3 | 1 | 1 | 1 | 10 |
|  | STN+PVP LFU STN_uni_on | 23.5 |  | 4.5 | 8 | 1 | 0 | 0 | 0 | 0 | 0 | 13.5 |  | 2 | 0 | 2 | 3 | 1 | 1 | 1 | 10 |
|  | STN+PVP LFU STN_bi_on | 18.5 | 0 | 3 | 6 | 0.5 | 0 | 0 | 0 | 0 | 0 | 9.5 |  | 2 | 0 | 2 | 2 | 1 | 1 | 1 | 9 |
| Patient2 | Pre_op | 9 | 0 | 0 | 0 | 6 | 0 | 0 | 0 | 0 | 0 | 6 |  | 0 | 0 | 0 | 0 | 2 | 1 | 0 | 3 |
|  | GPi 6m | 3 | 0 | 0 | 0 | 2 | 0 | 0 | 0 | 0 | 0 | 2 |  | 0 | 0 | 0 | 0 | 0 | 0 | 1 | 1 |
|  | GPi LFU | 18.5 | 8 | 0 | 0 | 4.5 | 0 | 0 | 0 | 0 | 0 | 12.5 |  | 0 | 0 | 0 | 0 | 2 | 2 | 2 | 6 |
|  | STN+PVP 1m STN_bi_on | 2 | 1 | 0 | 0 | 1 | 0 | 0 | 0 | 0 | 0 | 2 |  | 0 | 0 | 0 | 0 | 0 | 0 | 0 | 0 |
|  | STN+PVP 6m STN_bi_on | 1.5 | 1 | 0 | 0 | 0.5 | 0 | 0 | 0 | 0 | 0 | 1.5 |  | 0 | 0 | 0 | 0 | 0 | 0 | 0 | 0 |
|  | STN+PVP LFU STN_bi_off | 12.5 | 8 | 0 | 0 | 1.5 | 0 | 0 | 0 | 0 | 0 | 9.5 |  | 0 | 0 | 0 | 0 | 2 | 0 | 1 | 3 |
|  | STN+PVP LFU STN_uni_on | 7.5 | 4.5 | 0 | 0 | 1 | 0 | 0 | 0 | 0 | 0 | 5.5 |  | 0 | 0 | 0 | 0 | 1 | 0 | 1 | 2 |
|  | STN+PVP LFU STN_bi_on | 1.5 | 1 | 0 | 0 | 0.5 | 0 | 0 | 0 | 0 | 0 | 1.5 |  | 0 | 0 | 0 | 0 | 0 | 0 | 0 | 0 |
|  |  |  |  |  |  |  |  |  |  |  |  |  |  |  |  |  |  |  |  |  |  |
| Patient3 | Pre_op | 11 | 0 | 0 | 0 | 8 | 0 | 0 | 0 | 0 | 0 | 8 |  | 0 | 0 | 0 | 0 | 1 | 1 | 1 | 3 |
|  | GPi 6m | 6 | 0 | 0 | 0 | 4 | 0 | 0 | 0 | 0 | 0 | 4 |  | 0 | 0 | 0 | 0 | 1 | 1 | 0 | 2 |
|  | GPi LFU | 9 | 0 | 0 | 0 | 6 | 0 | 0 | 0 | 0 | 0 | 6 |  | 0 | 0 | 0 | 0 | 1 | 1 | 1 | 3 |
|  | STN+PVP 1m STN_bi_on | 1 | 0 | 0 | 0 | 1 | 0 | 0 | 0 | 0 | 0 | 1 |  | 0 | 0 | 0 | 0 | 0 | 0 | 0 | 0 |
|  | STN+PVP 6m STN_bi_on | 0.5 | 0 | 0 | 0 | 0.5 | 0 | 0 | 0 | 0 | 0 | 0.5 |  | 0 | 0 | 0 | 0 | 0 | 0 | 0 | 0 |
|  | STN+PVP LFU STN_bi_off | 1.5 | 0 | 0 | 0 | 1.5 | 0 | 0 | 0 | 0 | 0 | 1.5 |  | 0 | 0 | 0 | 0 | 0 | 0 | 0 | 0 |
|  | STN+PVP LFU STN_uni_on | 2 | 0 | 0 | 0 | 2 | 0 | 0 | 0 | 0 | 0 | 1.5 |  | 0 | 0 | 0 | 0 | 0 | 0 | 0 | 0 |
|  | STN+PVP LFU STN_bi_on | 0.5 | 0 | 0 | 0 | 0.5 | 0 | 0 | 0 | 0 | 0 | 0.5 |  | 0 | 0 | 0 | 0 | 0 | 0 | 0 | 0 |
|  |  |  |  |  |  |  |  |  |  |  |  |  |  |  |  |  |  |  |  |  |  |
|  |  |  |  |  |  |  |  |  |  |  |  |  |  |  |  |  |  |  |  |  |  |
| Patient4 | Pre_op | 5 | 0 | 0 | 0 | 3 | 0 | 0 | 0 | 0 | 0 | 3 |  | 0 | 0 | 0 | 0 | 1 | 1 | 0 | 2 |
|  | GPi 6m | 0.5 | 0 | 0 | 0 | 0.5 | 0 | 0 | 0 | 0 | 0 | 0.5 |  | 0 | 0 | 0 | 0 | 0 | 0 | 0 | 0 |
|  | GPi LFU | 6.5 | 0 | 0 | 0 | 4.5 | 0 | 0 | 0 | 0 | 0 | 4.5 |  | 0 | 0 | 0 | 0 | 1 | 1 | 0 | 2 |
|  | STN+PVP 1m STN_bi_on | 1 | 0 | 0 | 0 | 1 | 0 | 0 | 0 | 0 | 0 | 1 |  | 0 | 0 | 0 | 0 | 0 | 0 | 0 | 0 |
|  | STN+PVP 6m STN_bi_on | 1 | 0 | 0 | 0 | 1 | 0 | 0 | 0 | 0 | 0 | 1 |  | 0 | 0 | 0 | 0 | 0 | 0 | 0 | 0 |
|  | STN+PVP LFU STN_bi_off | 5 | 0 | 0 | 0 | 4 | 0 | 0 | 0 | 0 | 0 | 4 |  | 0 | 0 | 0 | 0 | 0 | 1 | 0 | 1 |
|  | STN+PVP LFU STN_uni_on | 1 | 0 | 0 | 0 | 1 | 0 | 0 | 0 | 0 | 0 | 1 |  | 0 | 0 | 0 | 0 | 0 | 0 | 0 | 0 |
|  | STN+PVP LFU STN_bi_on | 1 | 0 | 0 | 0 | 1 | 0 | 0 | 0 | 0 | 0 | 1 |  | 0 | 0 | 0 | 0 | 0 | 0 | 0 | 0 |
|  |  |  |  |  |  | 0 | 0 | 0 | 0 | 0 | 0 | 0 |  |  |  |  |  |  |  |  |  |
|  |  |  |  |  |  |  |  |  |  |  |  |  |  |  |  |  |  |  |  |  |  |
| Patient5 | Pre_op | 19 | 6 | 6 | 4 |  | 0 | 0 | 0 | 0 | 0 | 16 |  | 1 | 0 | 0 | 0 | 1 | 1 | 0 | 3 |
|  | GPi 6m | 5 | 1 | 1 | 2 | 0 | 0 | 0 | 0 | 0 | 0 | 4 |  | 1 | 0 | 0 | 0 | 0 | 0 | 0 | 1 |
|  | GPi LFU | 27 | 8 | 8 | 6 | 0 | 0 | 0 | 0 | 0 | 0 | 22 |  | 2 | 0 | 0 | 0 | 2 | 1 | 0 | 5 |
|  | STN+PVP 1m STN_bi_on | 6 | 1 | 2 | 2 | 0 | 0 | 0 | 0 | 0 | 0 | 5 |  | 1 | 0 | 0 | 0 | 0 | 0 | 0 | 1 |
|  | STN+PVP 6m STN_bi_on | 6 | 1 | 2 | 2 | 0 | 0 | 0 | 0 | 0 | 0 | 5 |  | 1 | 0 | 0 | 0 | 0 | 0 | 0 | 1 |
|  | STN+PVP LFU STN_bi_off | 14 | 4 | 6 | 2 | 0 | 0 | 0 | 0 | 0 | 0 | 12 |  | 1 | 0 | 0 | 0 | 0 | 1 | 0 | 2 |
|  | STN+PVP LFU STN_uni_on | 11 | 4 | 3 | 2 | 0 | 0 | 0 | 0 | 0 | 0 | 9 |  | 1 | 0 | 0 | 0 | 0 | 1 | 0 | 2 |
|  | STN+PVP LFU STN_bi_on | 5 | 1 | 1 | 2 | 0 | 0 | 0 | 0 | 0 | 0 | 4 |  | 1 | 0 | 0 | 0 | 0 | 0 | 0 | 1 |
|  |  |  |  |  |  | 0 | 0 | 0 | 0 | 0 | 0 | 0 |  |  |  |  |  |  |  |  |  |
|  |  |  |  |  |  |  |  |  |  |  |  |  |  |  |  |  |  |  |  |  |  |
| Patient6 | Pre_op | 34 | 0 | 1 | 9 | 0 | 6 | 6 | 0 | 1 | 1 | 22 |  | 1 | 2 | 1 | 3 | 1 | 1 | 1 | 10 |
|  | GPi 6m | 14 | 0 | 1 | 3 | 0 | 2 | 4 | 0 | 0 | 0 | 10 |  | 1 | 1 | 1 | 1 | 0 | 0 | 0 | 4 |
|  | GPi LFU | 51 | 0 | 3 | 4 | 4 | 12 | 12 | 0 | 2 | 2 | 35 |  | 2 | 2 | 2 | 1 | 2 | 2 | 1 | 12 |
|  | STN+PVP 1m STN_bi_on | 28 | 0 | 1 | 2 | 1 | 9 | 9 | 0 | 0 | 0 | 22 |  | 1 | 1 | 1 | 1 | 1 | 1 | 0 | 6 |
|  | STN+PVP 6m STN_bi_on | 22.5 | 0 | 1 | 3 | 0.5 | 6 | 6 | 0 | 0 | 0 | 16.5 |  | 1 | 1 | 1 | 1 | 1 | 1 | 0 | 6 |
|  | STN+PVP LFU STN_bi_off | 31 | 0 | 4 | 4 | 1 | 8 | 8 | 0 | 0 | 0 | 25 |  | 1 | 1 | 1 | 1 | 1 | 1 | 0 | 6 |
|  | STN+PVP LFU STN_uni_on | 29.5 | 0 | 3 | 4 | 0.5 | 8 | 8 | 0 | 0 | 0 | 23.5 |  | 1 | 1 | 1 | 1 | 1 | 1 | 0 | 6 |
|  | STN+PVP LFU STN_bi_on | 17.5 | 0 | 1 | 2 | 0.5 | 4 | 4 | 0 | 0 | 0 | 11.5 |  | 1 | 1 | 1 | 1 | 1 | 1 | 0 | 6 |

Supplementary Table 3. Raw data of SF-36 scores

| SF36 subscale | Paytient NO | Patient 1 | Patient 2 | Patient 3 | Patient 4 | Patient 5 | Patient 6 |
| --- | --- | --- | --- | --- | --- | --- | --- |
| General health | pre-GPi | 15 | 30 | 20 | 30 | 15 | 20 |
|  | GPi 6m | 70 | 70 | 45 | 65 | 40 | 60 |
|  | GPi LFU | 15 | 25 | 20 | 15 | 15 | 30 |
|  | STN+PVP 1m | 45 | 55 | 35 | 45 | 40 | 60 |
|  | STN+PVP 6m | 70 | 70 | 75 | 65 | 65 | 60 |
|  | STN+PVP 12m | 75 | 75 | 75 | 65 | 65 | 60 |
| Physical function | pre-GPi | 20 | 45 | 50 | 50 | 50 | 0 |
|  | GPi 6m | 80 | 70 | 75 | 95 | 95 | 45 |
|  | GPi LFU | 20 | 10 | 50 | 45 | 50 | 0 |
|  | STN+PVP 1m | 80 | 70 | 75 | 95 | 100 | 45 |
|  | STN+PVP 6m | 100 | 50 | 90 | 100 | 100 | 50 |
|  | STN+PVP 12m | 100 | 70 | 100 | 100 | 100 | 50 |
| Role physical | pre-GPi | 0 | 0 | 100 | 25 | 0 | 0 |
|  | GPi 6m | 0 | 0 | 100 | 100 | 100 | 0 |
|  | GPi LFU | 0 | 0 | 100 | 25 | 0 | 0 |
|  | STN+PVP 1m | 0 | 0 | 100 | 100 | 100 | 0 |
|  | STN+PVP 6m | 100 | 0 | 100 | 100 | 100 | 0 |
|  | STN+PVP 12m | 100 | 0 | 100 | 100 | 100 | 0 |
| Role emotional | pre-GPi | 0 | 0 | 33.333333 | 33.333333 | 0 | 0 |
|  | GPi 6m | 33.333333 | 33.333333 | 66.666667 | 33.333333 | 66.666667 | 33.333333 |
|  | GPi LFU | 0 | 0 | 33.333333 | 33.333333 | 0 | 0 |
|  | STN+PVP 1m | 33.333333 | 33.333333 | 100 | 33.333333 | 66.666667 | 33.333333 |
|  | STN+PVP 6m | 66.666667 | 66.666667 | 100 | 100 | 100 | 66.666667 |
|  | STN+PVP 12m | 100 | 66.666667 | 100 | 100 | 100 | 66.666667 |
| Social functional | pre-GPi | 25 | 50 | 25 | 25 | 0 | 0 |
|  | GPi 6m | 50 | 50 | 50 | 50 | 50 | 25 |
|  | GPi LFU | 25 | 25 | 25 | 25 | 25 | 0 |
|  | STN+PVP 1m | 50 | 50 | 50 | 75 | 75 | 25 |
|  | STN+PVP 6m | 75 | 75 | 75 | 100 | 100 | 50 |
|  | STN+PVP 12m | 75 | 75 | 75 | 100 | 100 | 50 |
| Body pain | pre-GPi | 31 | 62 | 52 | 52 | 52 | 52 |
|  | GPi 6m | 64 | 62 | 74 | 74 | 74 | 74 |
|  | GPi LFU | 31 | 41 | 62 | 62 | 52 | 52 |
|  | STN+PVP 1m | 64 | 62 | 84 | 74 | 74 | 74 |
|  | STN+PVP 6m | 74 | 84 | 84 | 74 | 74 | 74 |
|  | STN+PVP 12m | 74 | 84 | 84 | 74 | 74 | 74 |
| Vitality | pre-GPi | 35 | 30 | 40 | 55 | 40 | 20 |
|  | GPi 6m | 60 | 45 | 55 | 65 | 65 | 55 |
|  | GPi LFU | 35 | 20 | 40 | 40 | 40 | 30 |
|  | STN+PVP 1m | 60 | 45 | 60 | 65 | 65 | 55 |
|  | STN+PVP 6m | 70 | 75 | 75 | 80 | 80 | 55 |
|  | STN+PVP 12m | 80 | 80 | 80 | 80 | 85 | 55 |
| Mental health | pre-GPi | 32 | 40 | 32 | 40 | 32 | 32 |
|  | GPi 6m | 48 | 76 | 32 | 88 | 72 | 56 |
|  | GPi LFU | 32 | 32 | 8 | 32 | 48 | 32 |
|  | STN+PVP 1m | 48 | 64 | 32 | 76 | 72 | 48 |
|  | STN+PVP 6m | 56 | 76 | 40 | 88 | 76 | 56 |
|  | STN+PVP 12m | 60 | 88 | 40 | 88 | 76 | 60 |


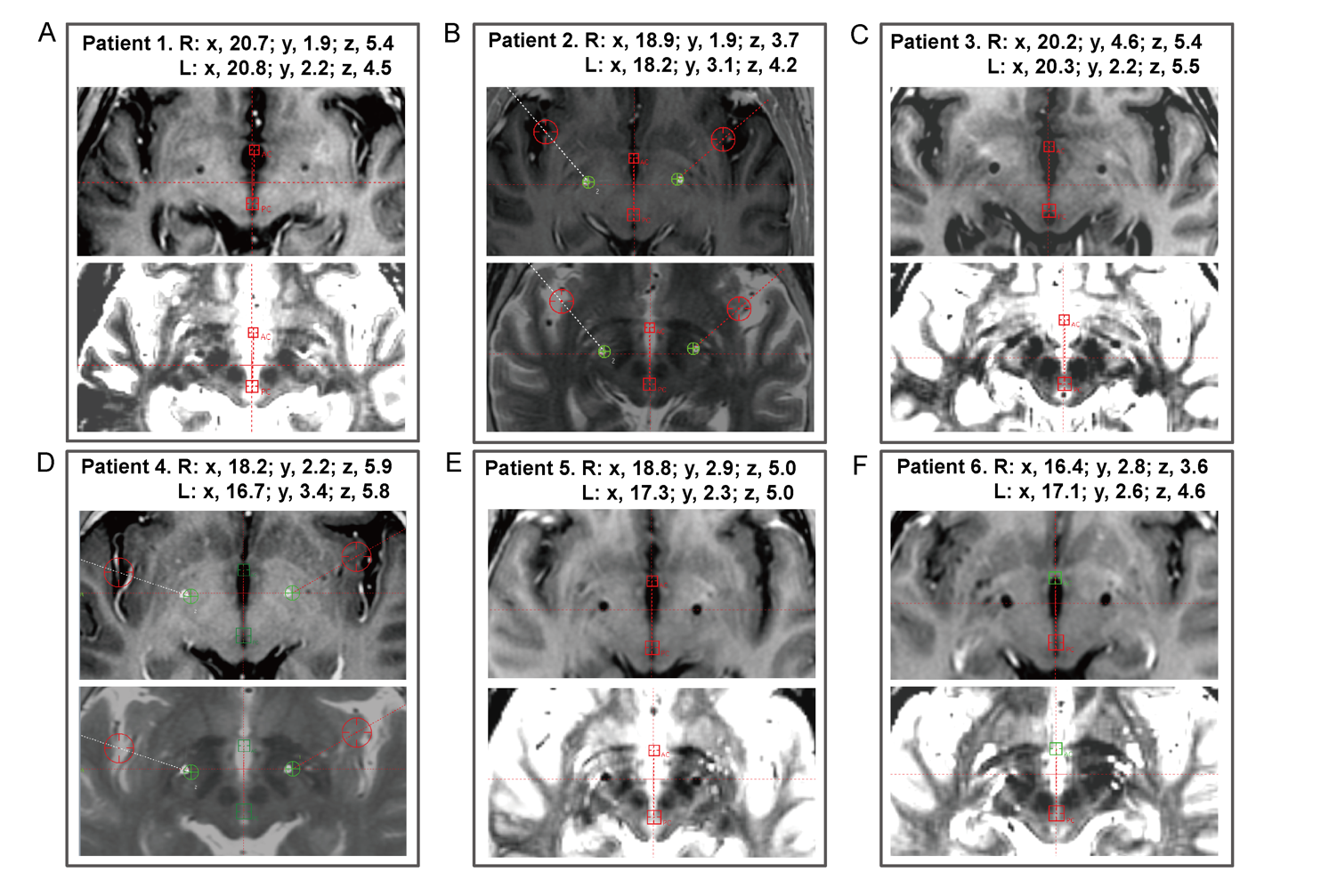


**Supplementary Figure 1.** Postoperative axial MRI image of different sequences shows the position of GPi electrodes of patient 1-6. The actual coordinates are also shown.
